# CancelRx case study: implications for clinic and community pharmacy work systems

**DOI:** 10.1186/s12913-023-10396-9

**Published:** 2023-12-06

**Authors:** Taylor L. Watterson, Jamie A. Stone, Peter C. Kleinschmidt, Michelle A. Chui

**Affiliations:** 1 University of Illinois Chicago College of Pharmacy, Chicago, IL USA; 2 University of Wisconsin-Madison School of Pharmacy, Madison, WI 53704 USA; 3 University of Wisconsin School of Medicine and Public Health, Madison, WI USA

**Keywords:** Health IT, Systems Approach, Case study, Pharmacy

## Abstract

**Background:**

Medication prescribing and discontinuation processes are complex and involve the patient, numerous health care professionals, organizations, health information technology (IT). CancelRx is a health IT that automatically communicates medication discontinuations from the clinic electronic health record to the community pharmacy dispensing platform, theoretically improving communication. CancelRx was implemented across a Midwest academic health system in October 2017. The health system also operates 15 outpatient community pharmacies.

**Objective:**

The goal of this qualitative study was to describe how both the clinic and community pharmacy work systems change and interact over time regarding medication discontinuations, before and after CancelRx implantation.

**Approach:**

Medical Assistants (*n* = 9), Community Pharmacists (*n* = 12), and Pharmacy Administrators (*n* = 3), employed by the health system were interviewed across 3-time periods between 2017 and 2018— 3-months prior to CancelRx implementation, 3-months after CancelRx implementation, and 9-months after CancelRx implementation. Interviews were audio recorded, transcribed, and conducted a hybrid analysis with deductive content analysis following the Systems Engineering Initiative for Patient Safety (SEIPS) framework and inductive analysis to capture additional codes and themes.

**Key results:**

CancelRx changed the medication discontinuation process at both clinics and community pharmacies. In the clinics, the workflows and medication discontinuation tasks changed over time while MA roles and clinic staff communication practices remained variable. In the pharmacy, CancelRx automated and streamlined how medication discontinuation messages were received and processed, but also increased workload for the pharmacists and introduced new errors.

**Conclusions:**

This study utilizes a systems approach to assess disparate systems within a patient network. Future studies may consider health IT implications for systems that are not in the same health system as well as assessing the role of implementation decisions on health IT use and dissemination.

**Supplementary Information:**

The online version contains supplementary material available at 10.1186/s12913-023-10396-9.

## Background

**Fig. 1:**
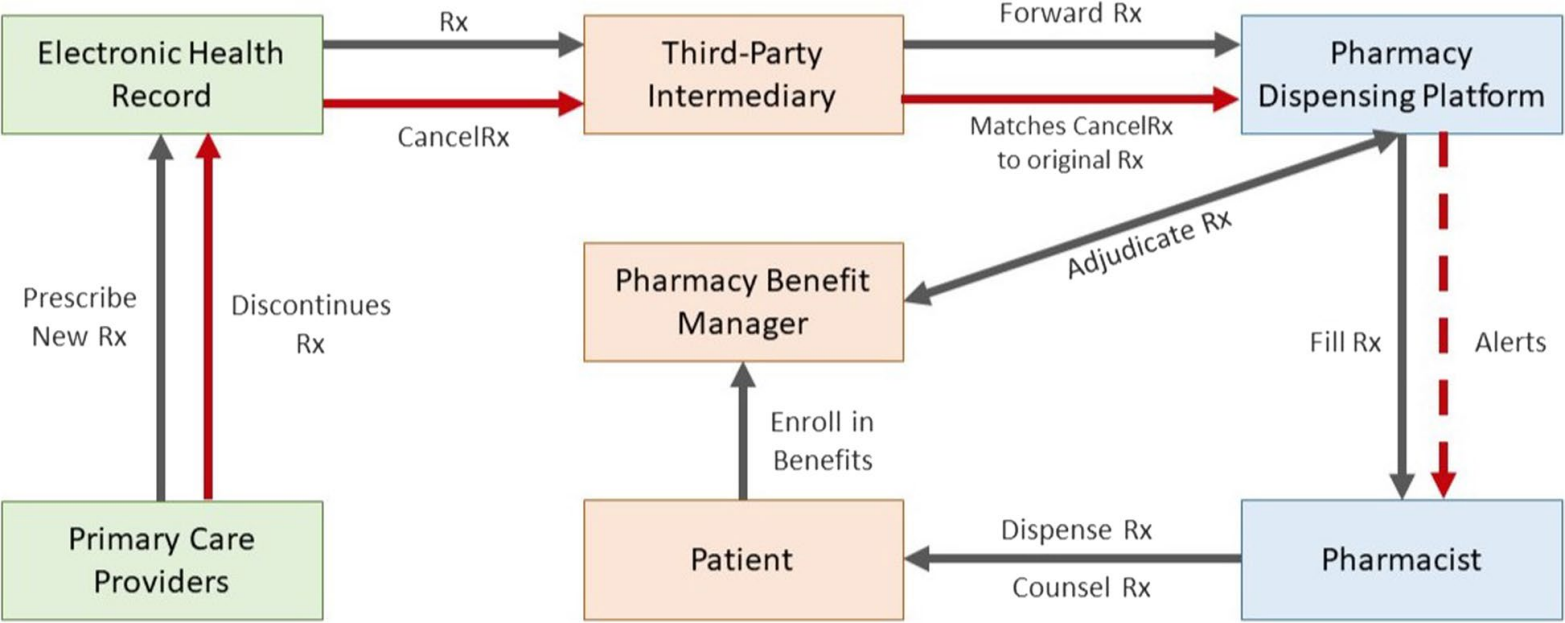
Simplified medication use process. When a patient attends their annual physical, their primary care provider (PCP) prescribes a new medication. The prescriber documents the prescription in the electronic health record (EHR) and the prescription is electronically sent to the pharmacy via a third-party intermediary, SureScripts. The pharmacy receives the prescription in their electronic dispensing platform, transcribes the information, and bills the patient’s insurance. The prescription is assessed by the pharmacist for accuracy and clinical appropriateness before filling and dispensing to the patient with appropriate consultation. CancelRx electronically communicates when medications are discontinued from the EHR to the pharmacy dispensing platform and alerts the pharmacist

Health care and health care delivery are complex due to the numerous people, organizations, and systems involved in the medication prescribing process [1, 2]. From inception to the patient’s bedside, prescribing a medication requires connectivity between numerous systems: clinic professionals, pharmacy professionals, clinic and pharmacy health information technology (health IT), health insurance payers (such as Pharmacy Benefit Mangers) patients, and their caregivers. This complex medication process is modeled in Fig. 1.

These multiple systems are important because they allow for specialization and expertise. However, when information is not shared or communicated effectively, there are risks to patient safety, especially when medications are stopped or discontinued [3–6].

### Medication discontinuation process

Medication discontinuation refers to when a healthcare provider elects to change or stop a patient’s medication regimen. Reasons for medication discontinuation often include: completion of therapy or clinical improvement; medication related adverse effects, drug interactions, or allergies; or change in therapy for an alternative dose or product [7].

When discontinuations are not communicated to pharmacies, medication records are inaccurate and pharmacies may inadvertently dispense medications that should have been stopped [3–6, 8]. One study found that 41% of the differences between clinic and pharmacy medication lists were attributable to discontinued medications [8]. This lack of communication and inaccurate pharmacy medication records makes patients vulnerable to errors and adverse drug events, especially when medications are dispensed that should have been discontinued for a serious drug reaction [4–6]. A 2003 study found that despite discontinuation in the clinic electronic health record (EHR), 5% of medications supposed to be stopped were later dispensed to patients at the pharmacy [6]. Health IT has the potential to improve communication between clinics and community pharmacies and address the discrepancies in medication lists across EHR and pharmacy dispensing software [9].

### CancelRx

A health IT functionality emerged within the last decade to facilitate the communication between clinics and pharmacies when medications are discontinued. CancelRx is a functionality that electronically deactivates a prescription at community pharmacy once the provider discontinues a medication at the clinic EHR [10, 11]. CancelRx has been implemented in many health care organizations and community pharmacies across the United States and its use is recommended by the National Council on Prescription Drug Programs SCRIPT guidelines, incorporated into the Department of Health and Human Services 2015 Edition Health Information Technology Certification Criteria, and CMS 2017 Stage 3 Meaningful Use EHR Criteria [10, 12, 13]. CancelRx is a non-propriety functionality that can be integrated into any EHR or pharmacy dispensing platform. A 2021 study reported an immediate, significant, and sustained increase in medication discontinuation communication between clinics and pharmacies after CancelRx implementation [14].

Similar to electronic prescriptions, CancelRx messages “travel” via the third-party platform, SureScripts, which links the clinic’s EHR to the pharmacy’s dispensing software. Because electronic prescriptions are also sent via SureScripts, CancelRx messages can automatically match to active prescriptions in the pharmacy dispensing software, alert pharmacy staff, and discontinue them—preventing future dispensing to patients.

Despite its already widespread reach, one significant barrier to complete adoption of CancelRx in every health system and community pharmacy is the concern about how it might impact workflow and workload [14, 15]. Research has demonstrated unintended negative consequences of health IT implementation—including workflow disruptions, additional tasks, and even patient safety concerns [16–21]. This study aimed to assess how a novel health IT, CancelRx, uniquely impacted and connected disparate systems as well as provide guidance for the design and implementation of future health IT.

## Objective

The goal of this qualitative study was to describe how clinic and community pharmacy work systems changed and communicated medication discontinuations over time. This study took a systems-based approach following the Systems Engineering Initiative for Patient Safety (SEIPS) framework.

## Methods

### Theoretical framework

Guiding the methodological approach was the Systems Engineering Initiative for Patient Safety (SEIPS) framework [22–24]. Within the SEIPS framework, a work system is comprised of five interconnected social and technical components (person, organization, technologies and tools, tasks, and [physical] environment) that impact how care is provided and the resulting patient outcomes [22].

### Setting

This study was conducted within UW Health, the integrated health system of the University of Wisconsin. UW Health serves more than 600,000 patients each year with six hospitals and 80 outpatient sites. UW Health also includes 15 outpatient community pharmacies which fill approximately 500,000 prescriptions annually. At the time of the study, UW Health utilized Epic (Epic Systems Inc., Verona, WI) as the EHR vendor and EnterpriseRx (McKesson, San Francisco, CA) as the pharmacy dispensing software vendor.

UW Health implemented CancelRx across the entire organization in October 2017 as part of a larger health IT upgrade. This study is specific to the UW Health system as well their clinic and pharmacy health IT vendors.

### Participant selection

The research team received approval by the University of Wisconsin-Madison Institutional Review Board prior to data collection. Data collection took place at 3 distinct time periods: at 3-months prior to CancelRx implementation, at 3-months after CancelRx implementation, and 9-months after CancelRx implementation. The time periods were selected to determine how clinic and pharmacy work systems and communication changed over time. Interviews 3-month prior to CancelRx gathered baseline information about work processes, workflow, and workload. Interviews 3-months post aimed to capture adoption and implementation of CancelRx in the clinic and pharmacy work systems. Finally, the focus of the interviews at 9-months post were to determine if and how the work systems were reshaped to facilitate maintenance and sustainment of effective CancelRx use [25].

Medical Assistant and Pharmacist sample sizes were determined a priori using research team expertise—estimating the amount of interviewed needed to be sufficient for saturation in light of time and budget constraints.

#### Medical assistant participants

With the approval and support of UW Health stakeholders, the research team presented the study during monthly staff meetings at 3 outpatient clinics. The clinics were pre-selected by UW Health leadership as able and likely to participate. The goal of the face-to-face convenience sampling approach was to recruit 3 medical assistants (MAs) at each of 3 clinics (9 total) to participate in the study. Once the study team received 3 participants per site, they stopped recruitment. MAs were chosen, as opposed to nurses, prescribers, or other clinic staff, because they were the individuals primarily responsible for contacting the pharmacy when a medication was discontinued pre-intervention. All 9 participants were retained throughout the study duration and participated at all 3 time periods.

#### Pharmacist participants

With the approval and support of UW Health stakeholders, the research team presented the study during site visits to 3 outpatient community pharmacies. The pharmacies were pre-selected by UW Health leadership as able and likely to participate. When time permitted, the research team would meet with several pharmacists to present the study and share contact information. Participants were invited to share their interest while the research team was on site or contact them via telephone. The goal of the face-to-face convenience sampling was to recruit 3 pharmacists at each of 3 pharmacies (9 total) to participate in the study. Over the course of 3 time periods, a total of 12 pharmacists were interviewed. While no participants outright refused at follow-up, non-participation reasons included moving to a new pharmacy within the organization, leaving the organization, and retirement. At 9-months post CancelRx, 3 UW Health pharmacy administrators were recruited via purposive sampling to capture organization wide decisions and outcomes related to CancelRx implementation.

### Data collection

The research team developed and piloted a semi-structured interview guide to ascertain how medication discontinuation orders were received, prioritized, addressed, and communicated, as well as facilitators and barriers to communicating medication discontinuation messages to pharmacies (MA) or discontinuing the messages in the EnterpriseRx software (pharmacy). Additionally, interview questions prompted the participant to discuss the impact of medication discontinuation messages on workflow, workload, and their perceived outcomes.

Interviews were conducted in a private room or location in the clinic or pharmacy, often an empty office, exam room, or consultation space; no non-participants were present. All interviews were conducted by either TW or KZ (acknowledgements). TW did not openly share they were a pharmacist with participants unless directly asked to minimize bias—e.g., a participant assuming the interviewer had pharmacy knowledge and excluding details or tailoring responses with a social desirability bias to avoid feeling evaluated or judged for clinical practices. Prior to starting the interview, participants were reminded of the purpose of the study and briefly reviewed on the study’s confidentiality agreement and plans to disseminate findings. Participants were able to stop or pause the interview at any time or skip any questions. Interviews ranged between 25 and 45 min. All interviews were audio recorded and transcribed verbatim via a professional agency. The research team did not utilize field notes during or after the interviews. While transcripts were not returned to participants for comment/correction, the research team often probed on statements made months prior for follow-up.

### Data analysis

The research team used NVivo 12 to analyze the semi-structured interview transcripts [26]. TW and JS conducted a hybrid qualitative analysis, first with deductive content analysis using the SEIPS framework [22–24, 27]. The research team created codes based on the SEIPS components (person, organization, technologies and tools, tasks, and [physical] environment) as well as sub-codes as identified in the literature [22, 23]. The research team also added inductive codes as needed. The completed codebook can be found in Additional file 1: Appendix A.

The two coders (TW and JS) established consensus and common mental models. They then independently coded several transcripts to establish interrater reliability (Cohen’s Kappa calculated via NVivo 12 and determined sufficient, > 0.80) [28–30]. They independently coded the remaining transcripts, and returned to review transcript sections that required additional discussion. After coding was complete, the researchers independently reviewed the findings and then met to aggregate the key codes into overarching themes to address the study aims.

While study sample size was determined a priori, TW and JS confirmed the data reached saturation—by the end of analysis, no new inductive codes or themes emerged and data became redundant [31].

## Results

While exact numbers of CancelRx messages are not known, in time spanning one year prior to CancelRx implementation to one-year after CancelRx implementation (October 2016 – October 2018), 354,690 medications were discontinued across all UW Health ambulatory clinics, and 369,509 medications were discontinued at the outpatient community pharmacies.

### The clinic system

A total of 9 medical assistants (MAs) were interviewed. On average respondents were 38 years old (SD 9.3) and had 14.2 years of experience working in a clinic setting (SD 8.9). All MA participants were female, white, and were not Hispanic or Latino.

The MA interviews yielded 2 key themes pertinent to how the clinic system changed over time: 1) workflow and tasks regarding medication discontinuations changed for clinic staff over time and 2) clinic staff roles, relationships, and communication patterns regarding medication discontinuations were variable and the variability persisted over time.

### Workflows and tasks regarding medication discontinuations changed for clinic staff over time

In general, there were several distinct workflows and accompanying tasks that occured when medications were discontinued at the clinic: first the medication was discontinued, second, the MA was made aware of the discontinuation, and third, the clinic communicated the medication discontinuation to the pharmacy. The tasks involved with these workflows changed over time, namely before and after CancelRx implementation.

#### Pre-CancelRx

MAs received notification of a medication discontinuation task via a message sent through the EHR called an “in-basket message” in a folder titled “medication discontinuation.” Although all the MAs within a clinic received all the in-basket messages as part of a centralized queue, most MAs reported only opening messages belonging to their assigned providers and patients. The in-basket message informed the MA that a patient’s medication was discontinued and that they should contact the patient’s community pharmacy. The MAs indicated that this process could become time consuming, especially if they were placed on hold at the pharmacy or transferred between staff members. Within the in-basket message, MAs were able to document notes regarding the encounter, such as whether they called the pharmacy, who they spoke to at the pharmacy, etc.

Interviews with MAs indicated there was variability in how these in-basket messages were handled, including several participants who were unfamiliar with the medication discontinuation folder and unaware of the in-basket messages informing them to contact the pharmacy. Most MAs reported that they did not call the pharmacy on every in-basket message. Most MAs utilized professional judgement or clinical decision making to determine when to contact the pharmacy and when to mark the messages as “Done” without contacting the pharmacy.

*It’s up to the provider if they discontinue it. If they do, it goes into a medication cancellation folder. And then it all depends if it’s a newer medication, and if it has refills, then we call the pharmacy and have them cancel the refills. Otherwise, if it’s a prescribed med that’s from two years ago, we just “done” it because there shouldn't be refills left on it.*

*-Medical Assistant 2, Clinic 2, Pre-CancelRx*

#### Post-CancelRx

CancelRx automatically communicated medication discontinuations between the clinic EHR and pharmacy dispensing software, potentially reducing the number instances where MAs were required to contact the pharmacy. However, medication discontinuation messages sent via CancelRx still appeared in the MA’s in-basket folder—informing them that the message had been communicated, as well as if and when the pharmacy discontinued the medication.

One MA reported that there were minimal differences between how the pre-CancelRx and CancelRx messages looked in the in-basket folder. Another MA stated that they knew which pharmacies were able to receive CancelRx messages, and when they saw an in-basket message directing them to contact a CancelRx pharmacy they immediately “done [the messages] out.” Other MAs interviewed were not aware of the CancelRx functionality, even 3-months after implementation.

At 9-months after CancelRx implementation, most MAs were aware of the CancelRx functionality. Some MAs also shared how their trust (or lack thereof) in the CancelRx technology influenced their practice—they continued to call on all in-basket messages regardless of CancelRx status or they no longer called on CancelRx messages because they were confident the pharmacy received the medication discontinuation.

*And some [messages] will say ‘cannot cancel’ and then list the reason. Or “please call the pharmacy.” So we call anyways. Then I call [the pharmacy] and sometimes what happens is that prescription is no longer there. They transferred it. If a patient transfers [a prescription] on their own, we don’t see that. And [the pharmacy] lets us know where it’s at, and then we can cancel it there.*

*-Medical Assistant 2, Clinic 1, 9-Months Post-CancelRx*

### Clinic staff roles, relationships and communication patterns regarding medication discontinuations were variable and the variability persisted over time

Within UW Health clinics, MAs were partnered with providers to see patients, document encounters, and complete administrative tasks. MA roles and communication patterns, as they related to medication discontinuations, were variable based on these physician partnerships. This variability existed prior to CancelRx implementation and persisted after its implementation.

#### Pre-CancelRx

Within the UW Health organization, there was ambiguity as to whether or not MAs were permitted to discontinue medications from a patient’s profile. Some MAs stated they were not allowed to discontinue medications from a patient’s profile under any circumstances while others reported that they were allowed to discontinue patient reported medications such as over-the-counter products or historical medications from outside UW Health. Other MAs indicated that they had agreements in place with their partnered provider that they were allowed to discontinue medications under certain conditions.

MAs utilized the EHR to communicate with their providers—updating the patient medication taking behavior or documenting findings within the encounter note. Some MAs also indicated that they would debrief with their providers in-person either before or after the provider saw the patient to review the medication list, discuss new, changed, or discontinued medications, and if they need to contact the pharmacy. Providers would also use the EHR to communicate and share tasks with MAs and clinic staff members via the in-basket messaging—alerting MAs to contact the patient’s pharmacy when a medication was discontinued.

*We’re not allowed to take it off the med list, so I’ll just click, ‘not taking’, and put it in my note and hope the doctor takes it off. [...] Only if it’s over the counter. Then I’ll take it off the med list, but if it’s a prescribed medicine, we’re not supposed to touch it.*

*-Medical Assistant 3, Clinic 2, Pre-CancelRx*

#### Post-CancelRx

After CancelRx implementation, variability still existed regarding whether MAs could remove medications from a patient’s EHR record. More MAs reported having agreements with their providers that allowed them to remove outdated or discontinued medications from the patient’s medication list.

MAs and providers still utilized the EHR functionality to communicate within the clinic when medications were changed or discontinued. MAs still received in-basket messages, even when CancelRx messages were sent and so the new functionality did not markedly change communication practices.

*Well, I'm not supposed to [discontinue medications]. But the providers that I work with, and I have an agreement. Because we had noticed that a lot of times on the patient’s medication list if I marked “not taking,” it just sits there. So the patient will come in once a month for follow-ups, and I have to go over that in the med list. “Okay, so you’re still not taking such-and-such medication?” Because the provider just never takes it out. So the providers that I work with have given me the okay to go ahead and discontinue medications, as long as, it’s based on what the patient says.*

*-Medical Assistant 3, Clinic 1, 3-Months Post-CancelRx*

### The pharmacy system

A total of 12 pharmacists and 3 pharmacy administrators (*n* = 15) were interviewed. The average age of the pharmacy staff members was 41.9 (SD 9.9). On average, pharmacy staff participants had 14.7 years of experience working in a pharmacy setting (SD 8.8). Over half of the participants were female (*n* = 10, 7%), almost all were white (*n* = 14. 9% white; 1 Asian), and none of the participants were Hispanic or Latino.

A medication was considered to be successfully discontinued when it was cancelled in both the clinic EHR and pharmacy dispensing platforms [14]. The pharmacist interviews elucidated themes regarding 1) changes to pharmacy medication discontinuation workflow and 2) changes to patient safety concerns over time.

### Workflows and tasks regarding medication discontinuations changed for pharmacy staff over time

During the pre-CancelRx interviews, pharmacists described their process for receiving discontinuation messages that were sent from the clinic. First, they received the message (either via phone call, voicemail, fax, notes on prescriptions, or patient report), then they navigated to the patient profile in the pharmacy dispensing software. Pharmacists reported that most discontinuation messages did not include a reason for discontinuation and that, occasionally, they investigated the discontinuation message further by reviewing the patient’s profile in the clinic EHR to read the provider’s notes. Finally, the pharmacists deactivated the prescription in the pharmacy dispensing software, cancelling future fills and refills of the medication, and removing it from the active medication list. The pharmacists sometimes documented the discontinuation via a note in the patient’s profile or informed other pharmacy staff members such as technicians or other pharmacists.

After CancelRx implementation, pharmacists reported that the main way they received discontinuation messages was electronically via CancelRx and that these CancelRx messages did not include a reason for why the prescription was discontinued. Pharmacists stated that if they wanted to determine the reason for cancellation, they would rely on their clinical judgement and make informed guesses based on the patients’ other medications or access the patient’s record in the EHR to see if the reason for discontinuation was documented in a provider note. Pharmacists reported that the CancelRx functionality was able to automatically find and match messages to linked prescriptions in the pharmacy’s dispensing profile and immediately discontinue the medication. Pharmacists were still required to review and attest to the CancelRx messages. However, once matched, they were unable to “un-discontinue” a CancelRx prescription.

*Just that it has been cancelled… that's really the only information we get from those [CancelRx] messages. And then if we want more information, we go into their Epic or HealthLink profile, verify that the medication has truly been discontinued, and then investigate a reason why if necessary.*

*-Pharmacist 5, Pharmacy 3, 9-months Post CancelRx*

Administration deemed reviewing and attesting to CancelRx messages was to be a “pharmacist only” task, as they determined it required clinical judgement and expertise.

*Our initial thought was we’d have the pharmacist [review and attest to the messages] for the first month and see if we could transition it to a technician . . . but after seeing all the nuances of whether it’s noise, we felt that it really needed to stay with the pharmacist. So, we haven’t really changed our process for it at all.*

*-Pharmacy Administrator 1, 9-month post CancelRx*

However, pharmacists mentioned that, in most situations, the task of attesting to the CancelRx messages could be given to the pharmacy technicians.

*Right now, [administration] wanted it as pharmacists only. I haven't heard if it's going to be a technician task. The [CancelRx messages] where it actually matches the prescription in the system, it's really not much, just the pharmacists going in and just hitting a button to say, “yep, this is canceled and matched it” sort of thing. And I think a technician could do that, because we can always look to see if it was canceled. Maybe for [CancelRx messages] that it doesn't exactly match it or if you have to search the patient's profile, it could be a pharmacist . . .*

*-Pharmacist 3, Pharmacy 3, 9-month post CancelRx*

### Medication discontinuation patient safety concerns changed over time

Pharmacists reported problems and patient safety concerns related to the medication discontinuation process both prior to, and after CancelRx implementation. The problems tended to fluctuate from not receiving enough discontinuation messages pre-CancelRx to receiving too many unimportant discontinuation messages post-CancelRx and other technological challenges.

#### Pre-CancelRx

Prior to CancelRx, pharmacists reported that medication discontinuation messages were generally not communicated reliably or consistently. This communication failure warranted medication safety concerns when patients were unsure of their medication regimens.

*There’s certainly been an opportunity for potential for harm. So, there’s an atenolol shortage right now, so patients are getting switched from atenolol to other beta blockers. So, in this case it was metoprolol. The patient wasn’t aware that they were being switched off it. She kept calling in her atenolol. She had been sent the metoprolol, and then she was sent a supply of atenolol. So now she’s getting a double beta blocker, potentially.*

*-Pharmacist 1, Pharmacy 3, Pre-CancelRx*

#### Post-CancelRx

After CancelRx implementation, pharmacists commented on the increased volume of CancelRx messages, particularly on the increased number of messages that were merely “nuisances.” Pharmacists indicated messages for acute or completed therapies were often unnecessary and warranted them to move quickly through the queue of CancelRx messages.

*Can I be embarrassing and say I don’t really even read them anymore? […] Well, it’s sad, but they’ve become more of a nuisance to me. I look to see how old the prescription is, what it’s for. It’s usually for a script that’s old or a non-maintenance med . . . or an antibiotic or something that, it doesn’t matter that it has been [discontinued]. The patient was on it short term. It should have been cleaned out of the profile ages ago, and it’s not appropriate to be sending us this message. […] It’s a very quick queue now. Where, in the beginning I was looking into the patient profile and going into HealthLink and trying to determine things, and now it’s just, oh, bogus, bogus, bogus. Get rid of it.*

*-Pharmacist 4, Pharmacy 1, 9-Months Post-CancelRx*

At 9-months post CancelRx, UW Health pharmacy administrators described the cost–benefit tradeoff discussions with utilizing CancelRx, including discussions about turning the functionality off completely. The administrators reported that the benefits of CancelRx, including patient safety efforts, outweighed the cost (i.e., the noise of excess messages). An administrator also shared that if CancelRx was turned off, they feared providers at both UW Health and other clinics would not know the functionality no longer worked, assume CancelRx messages were received at the pharmacy, and not communicate medication discontinuations via other means—thus posing more of a safety risk than pre-CancelRx practices.

*We ran into the challenges with billing as well as the noise. And then also the concern about what’s our responsibility once we receive these messages? But ultimately, in the end, the pharmacists felt that there was still value in getting these messages and that they’ve had some good catches out of it. So, we stuck with it and continue to stick with it. And we felt strongly that the clinical piece trumped the operational piece. So it would have a lot easier for us to turn it off and pretend we don’t know, and it’s fine. But we knew that wasn’t the right thing to do, so we stuck with the clinical aspect even though it’s more busy work for IT. It’s more busy work for fiscal, and it might mean some bunch of false negatives we’re looking into. But for the few good ones, it was worth doing.*

*-Pharmacy Administrator 1, 9-month post CancelRx*

## Discussion

MA and pharmacist interviews identified changes that occurred in two disparate work systems—clinic and community pharmacies—when the same CancelRx functionality was implemented.

### Role of technology in linking two separate systems

CancelRx had markedly different effects on the clinic and the pharmacy systems. For the MAs in the clinic setting, the medication discontinuation workload and tasks were slightly reduced after the implementation of CancelRx. Meanwhile, for the pharmacists in the community pharmacy, the workload was markedly increased. In this sense, the burden of the process was transferred from the clinic MA staff to the pharmacist with the creation of new work and tasks.

While some MAs were unaware that CancelRx was implemented, pharmacists were calling CancelRx messages “nuisances” and administrators considered turning off the functionality completely. The case of CancelRx demonstrates how when two systems are linked, changes in one system may lead to unintended consequences in the other.

Administrators, executives, and researchers should consider other external and linked systems when implementing new technologies or services. As part of the patient journey, these unintended consequences may lead to patient safety vulnerabilities or negative outcomes [16–19]. Due to the large volume of CancelRx messages, pharmacists may have been de-sensitized to the non-useful messages (CancelRx messages for expired prescriptions or acute fills), a phenomenon known as alert fatigue [32] Overwhelmed and fatigued, these pharmacists may have missed potentially crucial or more severe discontinuation messages that required complete attention. Identifying these vulnerabilities prior to turning on CancelRx may assist in making thoughtful implementation decisions to avoid alert fatigue risks. For example, setting system defaults to not automatically initiate CancelRx messages, only sending when prompted, and appropriately educating providers of the functionality. Similarly, CancelRx messaging may be set up to automatically send when specific reasons for discontinuation are selected, once again informing clinic staff of the need to accurately select reasons when discontinuing medications.

The case of CancelRx exemplifies the role for human factors engineering and other systems-based strategies such as proactive risk assessment when implementing novel technology, to consider not only unintended consequences within the system, but also in other externally linked systems that affect patient safety [33]. At Johns Hopkins, an interdisciplinary team conducted a proactive risk assessment as well as usability and pilot testing prior to CancelRx implementation [15] They identified strategies to mitigate risks when implementing CancelRx including adding the reason for discontinuation to the CancelRx message and considering whether all prior prescriptions should be discontinued. While these strategies were not evaluated for their impact on patient safety, the Johns Hopkins team illustrated how organizations can operationalize safety recommendations set by the Office of the National Coordinator for Health Information Technology including: multiple stakeholder involvement, identification of ideal workflows, and pilot and incremental testing [34–36].

CancelRx illustrated the importance of considering unintended consequences of novel health IT in disparate systems within the same patient network. When assessing and mitigating unintended consequences of novel health IT, the tendency may be to focus on the identifying vulnerabilities in the functionality and usability of the technology itself [37, 38]. However, the policies and culture surrounding the technology use, are also crucial to consider.

CancelRx implementation both influenced and was influenced by UW Health social systems. In the clinic, CancelRx was used differently depending on the role of the staff member. Providers, and sometimes MAs when permitted, were sending the CancelRx messages when discontinuing medications from the patient’s EHR record. MAs were responsible for following up or communicating medication discontinuation messages in the event of CancelRx failure. MAs reported instances when providers would not discontinue medications from the patient’s EHR profile. Some cited frustrations while others detailed agreements with their partnered providers to remove discontinued medications from the patient’s record. Assessing the appropriateness of UW Health’s decision and provider practices regarding MA discontinuation of medications is beyond the scope of this study, however; CancelRx messages are not sent to the pharmacy if they are not discontinued from the EHR medication list.

### Limitations

The study captured data from clinics and community pharmacies located within one organization; the health system’s outpatient community pharmacies also had access to the clinic EHR data which is not common for all community pharmacies, such as retail or independent establishments.

## Conclusion

CancelRx was implemented across UW Health in October 2017 and changed the medication discontinuation process at both clinics and community pharmacies. In the clinics, the workflows and medication discontinuation tasks changed over time, while MA roles and clinic staff communication practices remained variable. In the pharmacy, CancelRx automated and streamlined how medication discontinuation messages were received and processed, but also increased workload for the pharmacists and introduced new errors.

This study utilizes a systems approach to assess disparate systems within a patient network. Future studies may consider health IT implications for systems that are not in the same health system as well as assessing the role of implementation decisions on health IT use and dissemination.

### Supplementary Information

**Additional file 1:** **Appendix A.** Codebook.

## Data Availability

The data underlying this article cannot be shared publicly for the privacy of individuals that participated in the study. Deidentified will be shared on reasonable request to the corresponding author.
