# Supplementary material for: CancelRx case study: implications for clinic and community pharmacy work systems
Source: BMC Health Serv Res. 2023 Dec 6;23:1360. doi: 10.1186/s12913-023-10396-9 (PMC10698877; doi:10.1186/s12913-023-10396-9)
Supplement: Supplementary file 1 — Additional file 1: Appendix A. Codebook. [file 12913_2023_10396_MOESM1_ESM.docx]

Appendix A: Codebook

| Name | Description |
| --- | --- |
| CancelRx Interactions | How CancelRx interacts with components of the sociotechnical work-system |
| CancelRx As Designed - Intended | How CancelRx was designed or intended to work, perform, or fit into the existing sociotechnical work systems |
| CancelRx DID NOT meet expectations | CancelRx did not meet the expectations for how it was designed or intended to work |
| CancelRx Implementation Decisions - Suggestions | Decisions made surrounding CancelRx implementation as well as suggestions if they were to do it again or for other organizations |
| Changes in communication patterns or practices | CancelRx hanged in communication patterns and practices |
| How CancelRx integrates in other HIT | How CancelRx integrates in current HIT [depends on current HIT[ |
| More or new work | CancelRx created more or new work |
| New types of errors or problems | CancelRx generated more or new errors and concerns to patient safety |
| Suggestions for CancelRx Redesign | Suggestions to CancelRx re-design, or changes to make it fit better into the sociotechnical systems |
| Changes in power structure or dynamics | CancelRx changes in the power structure |
| Cost - Benefit Tradeoff | Discussion of cost-benefit tradeoff of CancelRx |
| Workarounds | ISTA > Social system mediates HIT use > Workarounds |
| Organization | Organization (clinic or pharmacy) factors |
| Coordination, collaboration and communication_Clinic_Post CancelRx | Coordination, collaboration and communication within the clinic and between clinic staff and their patients after CancelRx implementation. Examples: Prescriber-Patient, Prescriber-Prescriber, Prescriber-MA, MA-Patient |
| Coordination, collaboration and communication_Clinic_Pre CancelRx | Coordination, collaboration and communication within the clinic and between clinic staff and their patients prior to CancelRx implementation. Examples: Prescriber-Patient, Prescriber-Prescriber, Prescriber-MA, MA-Patient |
| Coordination, collaboration and communication_ClinPharm_Post CancelRx | Coordination, collaboration and communication between clinic and pharmacy staff and work systems after CancelRx implementation |
| Coordination, collaboration and communication_ClinPharm_Pre CancelRx | Coordination, collaboration and communication between the clinic and pharmacy staff and work systems before CancelRx implementation |
| Coordination, collaboration and communication_Pharmacy_Post CancelRx | Coordination, collaboration and communication within the pharmacy and between pharmacy staff and their patients after CancelRx. Examples: pharmacist-patient, pharmacist-technician, technician-patient, technician-technician |
| Coordination, collaboration and communication_Pharmacy_Pre CancelRx | Coordination, collaboration and communication within the pharmacy and between pharmacy staff and their patients prior to CancelRx. Examples: pharmacist-patient, pharmacist-technician, technician-patient, technician-technician |
| Organizational culture_MED DISC_Post CancelRx | Organizational culture and patient safety culture (including policies and procedures) after CancelRx implementation |
| Organizational culture_MED DISC_Pre CancelRx | Organizational culture and patient safety culture (including policies and procedures) prior to CancelRx implementation |
| Staffing_MA_Post CancelRx | MA Work schedules (number of staff members working at clinic) after CancelRx implementation |
| Staffing_MA_Pre CancelRx | MA Work schedules (number of staff members working at clinic before CancelRx implementation |
| Staffing_NURSE_Post CancelRx | Nurse Work schedules (number of staff members working at clinic) after CancelRx implementation |
| Staffing_NURSE_Pre CancelRx | Nurse Work schedules (number of staff members working at clinic before CancelRx implementation |
| Staffing_PRESCRIBER_Post CancelRx | Prescriber Work schedules (number of staff members working at clinic) after CancelRx implementation |
| Staffing_PRESCRIBER_Pre CancelRx | Prescriber Work schedules (number of staff members working at clinic before CancelRx implementation |
| Staffing_Pharmacy_Post CancelRx | Pharmacy Work schedules (number of staff members working at clinic) after CancelRx implementation |
| Staffing_Pharmacy_Pre CancelRx | Pharmacy Work schedules (number of staff members working at clinic before CancelRx implementation |
| Person |  |
| Motivation, needs and beliefs_Post CancelRx | Motivation and needs / psychological characteristics related to medication discontinuation after CancelRx |
| Motivation, needs and beliefs_Pre CancelRx | Motivation and needs / Psychological characteristics related to medication discontinuation before CancelRx |
| Skills, knowledge, beliefs_MED DISC_Post CancelRx | Education, skills, knowledge, motivation, needs, and beliefs related to medication discontinuation (i.e. don't communicate a message because know that is an antibiotic [skill] and does not think that necessary to communicate [belief]) after CancelRx implementation |
| Skills, knowledge, beliefs_MED DISC_Pre CancelRx | SEIPS > work system or structure > Person > education, skills, knowledge, motivation, needs, and beliefs related to medication discontinuation (i.e. don't communicate a message because know that is an antibiotic [skill] and does not think that necessary to communicate [belief]) prior to CancelRx implementation |
| Physical Environment |  |
| Physical environment_Post CancelRx | Layout, noise, lighting, temperature, humidity, air quality after CancelRx implementation |
| Physical environment_Pre CancelRx | Layout, noise, lighting, temperature, humidity, air quality prior to CancelRx |
| Tasks |  |
| Number of MED DISC_Post CancelRx | Number of medication discontinuation messages after CancelRx |
| Number of MED DISC_Pre CancelRx | Number of medication discontinuation messages prior to CancelRx |
| Number of prescriptions_Post CancelRx | Number of prescriptions processed over a period of time at the pharmacy after CancelRx |
| Number of prescriptions_Pre CancelRx | Number of prescriptions processed over a period of time at the pharmacy prior to CancelRx |
| Role and Autonomy_Post CancelRx | Autonomy, job control, and participation regarding medication discontinuation after CancelRx |
| Role and Autonomy_Pre CancelRx | Autonomy, job control, and participation regarding medication discontinuation prior to CancelRx CancelRx |
| Tasks and workflow_MED DISC_Post CancelRx | Medication discontinuation workflow after CancelRx |
| Tasks and workflow_MED DISC_Pre CancelRx | Medication discontinuation workflow pre CancelRx |
| Tasks and workflow_NOT MED DISC_Post CancelRx | Variety of tasks; job content, job demands not related to medication discontinuation after CancelRx |
| Tasks and workflow_NOT MED DISC_Pre CancelRx | Variety of tasks; job content, job demands not related to medication discontinuation prior to CancelRx |
| Technologies and Tools |  |
| Technologies and tools_CANCELRX_Post CancelRx | Technologies and tools regarding CancelRx after CancelRx |
| Technologies and tools_NOT MED DISC_Post CancelRx | Technologies and tools not related to medication discontinuation after CancelRx Examples: various information technologies (EHR, CPOE, bar coding, pharmacy dispensing platofrm); medical devices |
| Technologies and tools_NOT MED DISC_Pre CancelRx | Technologies and tools not related to medication discontinuation prior to CancelRx Examples: various information technologies (EHR, CPOE, bar coding, pharmacy dispensing platofrm); medical devices |
| Technology and Tools_MED DISC not CancelRx_Pre CancelRx | Technologies and tools related to medication discontinuation but are NOT CancelRx, prior to CancelRx implementation |
| Pharmacy Administration |  |
| Administration Communication - Coordination | Administrator communicates and coordinates with other pharmacy team members or other pharmacy administrators |
| Administrator Role | Administrator role in CancelRx, including decisions, gaining organization support, and implementation |
